# Toxicity Tests: “Inert” and Active Ingredients

**DOI:** 10.1289/ehp.113-a657c

**Published:** 2005-10

**Authors:** Michael H. Surgan

**Affiliations:** Environmental Protection Bureau New York State Attorney General’s Office, New York, New York, E-mail: michael.surgan@oag.state.ny.us

The findings of Richard et al. (2005) are an important addition to our understanding that the health and environmental effects of formulated pesticide products are not fully reflected in tests conducted on the active ingredient(s) alone. It has been long known that the adjuvants (commonly and misleadingly called “inert” ingredients) may be toxic and may enhance or supplement the toxic effects of the active pesticidal ingredient.

In the case of glyphosate-containing products, this phenomenon was well demonstrated in the data submitted to the (EPA) by the registrant (Monsanto), and summarized by the U.S. EPA in the Reregistration Eligibility Document (RED) for glyphosate (U.S. EPA 1993). For example, based on the registrant’s own tests of acute toxicity to freshwater fish, the U.S. EPA classified technical grade glyphosate as “slightly toxic” to “practically non-toxic” and formulated products ranged from “moderately toxic” to “practically non-toxic.” Tested alone, the surfactant adjuvant (identified as “inert”) was “highly toxic” to “slightly toxic.” Similar differences were reported in tests of acute toxicity to freshwater invertebrates.

Based in part on the data in the glyphosate RED (U.S. EPA 1993), the New York State Attorney General’s office successfully pursued an action against Monsanto in 1996 (Attorney General of the State of New York 1996). At that time, Monsanto was making advertising claims about the toxicity of the Roundup products based on data from tests on the active ingredient alone. Such claims are scientifically unfounded and inherently deceptive. The Attorney General’s action was facilitated by the availability of at least some limited information about the inert ingredients and their toxicity. That same sort of information enabled Richard et al. (2005) to conduct their study.

Unfortunately, that is not always the case, and for many pesticide products, little or no information about the identity of inert ingredients is publicly available. Registrants are generally required to conduct acute toxicity tests on formulated products, but they traditionally conduct chronic toxicity tests on the active ingredient alone. Even when formulated products are tested, the identity of inert ingredients is rarely revealed in the open literature, publicly available regulatory documents, or product labels. Therefore, independent research is stymied, and the public is ill-informed in the marketplace.

## References

[b1-ehp0113-a0657c] Attorney General of the State of New York 1996. In the Matter of Monsanto Company, Respondent. Assurance of Discontinuance Pursuant to Executive Law § 63(15). New York:Attorney General of the State of New York, Consumer Frauds and Protection Bureau, Environmental Protection Bureau.

[b2-ehp0113-a0657c] Richard S, Moslemi S, Sipahutar H, Benachour N, Seralini G-E (2005). Differential effects of glyphosate and Roundup on human placental cells. Environ Health Perspect.

[b3-ehp0113-a0657c] U.S. EPA 1993. Reregistration Eligibility Decision (RED). Glyphosate. EPA-738-R-93-014. Washington, DC:U.S. Environmental Protection Agency. Available: http://cfpub.epa.gov/oppref/rereg/status.cfm?show=rereg [accessed 1 September 2005].

